# The Differential Reactive Oxygen Species Production of Tear Neutrophils in Response to Various Stimuli In Vitro

**DOI:** 10.3390/ijms222312899

**Published:** 2021-11-29

**Authors:** Yutong Jin, Brian Dixon, Lyndon Jones, Maud Gorbet

**Affiliations:** 1 School of Optometry and Vision Science, University of Waterloo, Waterloo, ON N2L 3G1, Canada; y46jin@uwaterloo.ca (Y.J.); lyndon.jones@uwaterloo.ca (L.J.); 2 Centre for Ocular Research and Education, University of Waterloo, Waterloo, ON N2L 3G1, Canada; 3 Department of Biology, University of Waterloo, Waterloo, ON N2L 3G1, Canada; bdixon@uwaterloo.ca; 4 Department of Systems Design Engineering, University of Waterloo, Waterloo, ON N2L 3G1, Canada

**Keywords:** tear neutrophils, ocular stress, oxidative burst, reactive oxygen species, NADPH oxidase, intracellular pathway

## Abstract

A large number of polymorphonuclear neutrophils (PMNs) invade the ocular surface during prolonged eye closure (sleep); these leukocytes are commonly referred as tear PMNs. PMNs contribute to homeostasis and possess an arsenal of inflammatory mediators to protect against pathogens and foreign materials. This study examined the ability of tear PMNs to generate reactive oxygen species (ROS), an essential killing mechanism for PMNs which can lead to oxidative stress and imbalance. Cells were collected after sleep from healthy participants using a gentle eye wash. ROS production in stimulated (phorbol-12-myristate-13-acetate (PMA), lipopolysaccharides (LPS) or N-Formylmethionyl-leucyl-phenylalanine (fMLP)) and unstimulated tear PMNs was measured using luminol-enhanced chemiluminescence for 60 min. A high level of constitutive/spontaneous ROS production was observed in tear PMNs in the absence of any stimulus. While tear PMNs were able to produce ROS in response to PMA, they failed to appropriately respond to LPS and fMLP, although fMLP-stimulated tear PMNs generated ROS extracellularly in the first three minutes. Higher ROS generation was observed in isolated tear PMNs which may be due to priming from the magnetic bead cell separation system. The differential responses of tear PMNs in ROS generation provide further evidence of their potential inflammatory roles in ocular complications involving oxidative stress.

## 1. Introduction

Polymorphonuclear neutrophils (PMNs), also known as neutrophils, are potent innate immune cells. PMNs play an essential role in protecting host tissues against foreign particles through oxidative bursts, degranulation, and phagocytosis, and possess many anti-inflammatory and pro-inflammatory responses. Like other immune cells, PMN populations have been shown to be heterogeneous, and can exhibit phenotypes distinct from blood-circulating PMNs in different tissues (such as in the mouth [1], placenta [2], lungs [3], and on the ocular surface [4]). A nocturnal-diurnal pattern has been observed in tear PMNs, the largest leukocyte population collected from the ocular surface. After prolonged eye closure, several thousand of PMNs can be collected from the ocular surface using a gentle wash, while collection during the day (open-eye conditions) often provides only a few hundred PMNs [4]. While this has not yet been fully elucidated, high levels of chemotactic molecules (e.g., interleukin-8 and complement products) detected in tears collected after sleep may explain the recruitment of PMNs at night [5]. Unlike blood PMNs, tear PMNs collected after more than 6 hr of sleep (i.e., closed-eye conditions) have upregulated expression of Mac-1 (CD11b, a cell adhesion and activation marker) and CD66b (a degranulation marker) and also exhibit a downregulation of L-selectin, a molecule reported to be shed upon activation or extravasation [4,6]. These observations suggest that tear PMNs collected after sleep may have already been activated following their arrival on the ocular surface due to the presence of inflammatory cytokines and foreign particles such as bacteria [6]. This hypothesis has been further supported by the presence of neutrophil extracellular traps (NETs) in the eye discharge [7]. Additionally, tear PMNs have been reported to be unable to upregulate or downregulate cell activation membrane receptors in response to various inflammatory stimuli in vitro [4,6]. However, very little is currently known about their ability to generate reactive oxygen species (ROS), with only one study assessing intracellular ROS production using flow cytometry at one specific time point and pointing towards a potential exhaustion or non-inflammatory phenotype due to the lack of response [4].

Oxidative burst, a potent killing mechanism in PMNs, has been investigated extensively in blood PMNs. Although ROS produced during the oxidative burst can eliminate foreign particles, excessive ROS production may also cause severe damage to surrounding tissues [8]. During the oxidative burst, nicotinamide adenine dinucleotide phosphate oxidase (NADPH oxidase) reduces molecular oxygen to superoxide anion ($O_{2}^{-}$) upon activation, leading to an increase in oxygen consumption by PMNs [8,9]. NADPH oxidases found in PMNs plasma membranes contribute to the extracellular production of superoxide anions, whereas NADPH oxidases in PMN granular membranes generate superoxide anions in an intracellular manner [10]. Complement fragment C5a, opsonized microorganisms, platelets activating factor (PAF), N-Formyl-L-methionyl-L-leucyl-L-phenylalanine (fMLP, a chemotactic peptide secreted by bacteria), lipopolysaccharides (LPS, fragments of gram-negative bacteria outer cell membranes), and phorbol-12-myristate-13-acetate (PMA) have been found to be efficient stimuli of the oxidative burst in PMNs [8]. The interaction between these stimuli and their corresponding receptors triggers a series of intracellular signaling pathways inducing different cell functional responses that have been well documented in blood PMNs [11].

To gain a better understanding of the role and function of tear PMNs in ocular homeostasis, this study investigated their ability to produce ROS in response to fMLP and LPS, which act via G protein-coupled receptors (GPCRs) and toll like receptors (TLRs), respectively, and PMA, which directly activates protein kinase C (PKC). ROS production was assessed by luminol-enhanced chemiluminescence, as it provides a kinetic measurement of ROS formation and can efficiently detect superoxide anions from both intra- and extracellular compartments [12,13]. ROS production in response to stimulus was measured directly in the cell collection from the closed-eye environment as well as following isolation of the tear PMNs population from the cell collection. While PMNs represent the largest population in the cell collection from the closed-eye environment, other leukocytes as well as a few dead corneal epithelial cells and occasionally goblet cells are also present. The expression of cell membrane receptors associated with cell activation was also assessed by flow cytometry following PMN isolation from the eye wash collection.

## 2. Results

**Figure 1:**
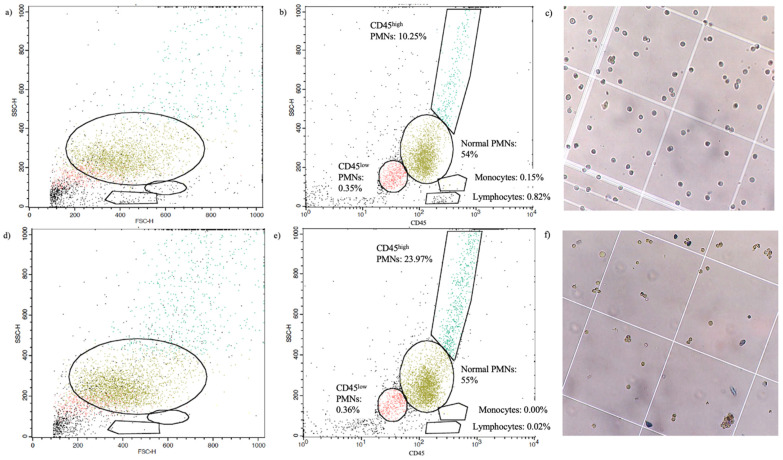
Identification of different cell populations using flow cytometry double-gating strategy in mixed tear PMNs (**a**–**c**) and MACS-isolated PMNs (**d**–**f**). (**a**,**d**) Side scatter versus forward scatter dot plots identifying leukocyte populations based on their size and cytoplasmic complexity. (**b**,**e**) Side scatter versus CD45 fluorescence (pan leukocyte marker) dot plot with CD45^+^ cells. Different cell populations have been identified along with their relative percentage. (**c**,**f**) Light microscopy images of mixed and MACS-isolated tear PMNs. Cell aggregates can be observed following isolation by the MACS column.

The eye wash collected from the closed-eye environment upon awakening contained different cell populations. Flow cytometry was used to identify cell populations based on their properties of forward and sideward size scatter and CD45 expression. The following populations were identified: PMNs (57.7 ± 7.7%), lymphocytes (1.5 ± 1.2%), monocytes (0.18 ± 0.18%), other CD45^+^ cells (20.2 ± 5.0%), the remainder of the events being ocular surface cells and debris. Within the 20% of CD45^+^ cell population, two subsets of population, CD45^low^ (~5%) and CD45^high^ (up to 10%), could be observed: expression of CD11b and CD66b on these CD45^+^ cells strongly suggest that these cells are a subpopulation of PMNs with different size scatters (Figure 1a,b). Experiments were also performed with tear PMNs that had been isolated from the eye wash collection using CD15^+^ positive selection with the MiniMACS column-based cell separation system (Miltenyi Biotec Inc., Auburn, CA, USA). PMNs in this purified fraction represented 88.6 ± 7.9% of the cell populations/events. When reporting and discussing results on ROS production, purified samples are referred as MACS-isolated tear PMNs, while the non-purified eye wash collection is referred to as mixed tear PMNs to reflect the presence of different cell populations in the eye wash while recognizing that PMNs represent the largest cell population and main contributor of ROS production of the cells present in the collection (epithelial cells, while able to generate ROS, are necrotic, and lymphocytes [14,15] and monocytes [16], which can also produce ROS, contributed to less than 2% of the collection).

### 2.1. ROS Production in LPS-Stimulated PMNs

**Figure 2:**
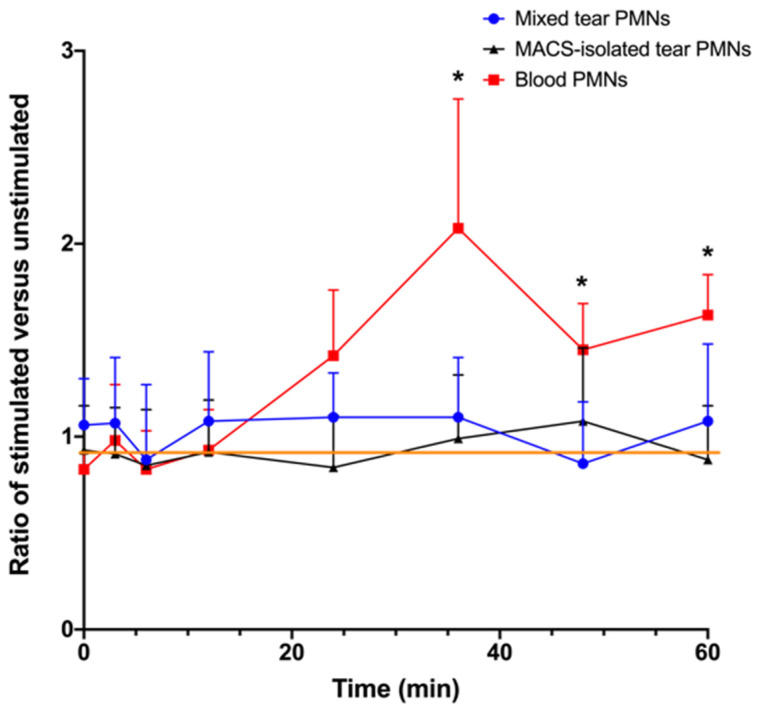
Changes in chemiluminescence (CL) of LPS-stimulated PMNs. Results are reported as ratio of LPS-stimulated PMNs CL emissions over control (unstimulated PMNs) CL emissions. A total of 50,000 cells were transferred to wells containing 1 mM luminol and HBSS followed by the addition of 2 μg/mL LPS. Generation of ROS was measured over time using the Cytation 5 at 37 °C. The orange line indicates a ratio of 1, meaning no difference between stimulated and unstimulated CL values. Values are presented as means ± standard deviations; mixed tear PMNs (n = 10–12 from 10 participants), MACS-isolated tear PMNs (n = 6 from 6 participants), and blood PMNs (n = 5 from 4 participants). * Significantly different from mixed and MACS-isolated tear PMNs (*p* ≤ 0.037).

As shown in Figure 2, LPS did not induce an oxidative burst in either the mixed tear PMNs or the MACS-isolated tear PMNs (CL ratio being close to 1). As expected, blood PMNs produced ROS from 12 min onwards; ROS production was significantly different between blood and tear PMNs (isolated or non-isolated) after 30 min (*p* ≤ 0.037).

The area under the curve (AUC) can be computed to reflect the quantity of ROS produced over 60 min. Both the absolute (for unstimulated and stimulated cells) and relative ROS production (using curves generated by calculating the ratio of stimulated versus unstimulated cells and activation ratio, as depicted in Figure 2) were calculated. As reported in Table 1, when considering absolute values, unstimulated mixed tear PMNs showed higher absolute ROS production as compared to both unstimulated and stimulated blood PMNs’ ROS production. However, as shown by the AUC activation ratio values, LPS-stimulated tear PMNs produced relatively less ROS as they failed to generate ROS in response to LPS. No significant differences were observed between groups (*p* > 0.05).

### 2.2. ROS Production in fMLP-Stimulated PMNs

**Figure 3:**
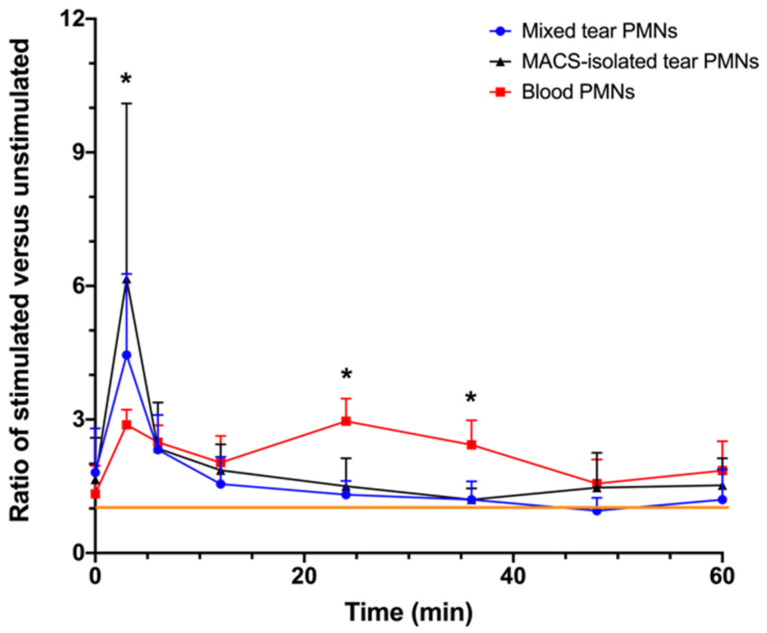
Changes in chemiluminescence (CL) of fMLP-stimulated PMNs. Results are reported as ratio of fMLP-stimulated CL emissions over control (unstimulated) sample CL emissions. A total of 50,000 cells were transferred to wells containing 1 mM luminol and HBSS, followed by the addition of 1.5 μM fMLP. Generation of ROS was measured over time using the Cytation 5 at 37 °C. The orange line indicates a ratio of 1, meaning no difference between stimulated and unstimulated CL values. Values are presented as means ± standard deviations; mixed tear PMNs (n = 14-16 from 16 participants), MACS-isolated tear PMNs (n = 10 from 8 participants), and blood PMNs (n = 5 from 4 participants). * Significantly different from tear PMNs and blood PMNs (*p* ≤ 0.02).

The kinetics of ROS production in fMLP-stimulated PMNs over 60 min are reported in Figure 3. At the 3 min time point, there was a transient peak of ROS production in fMLP-stimulated mixed and MACS-isolated tear PMNs. ROS production in tear PMNs at 3 min was significantly higher than that of blood PMNs (*p* ≤ 0.02). The large CL ratio variations in the tear PMNs noted at 3 min may be due to individual differences in ROS production or the fact that the stimulus was added to the samples manually, which might have induced some variations in the time of the rapid response elicited by fMLP. As expected and previously reported by others [17], a second peak in ROS production was observed in blood PMNs around 24 min (ROS production was significantly different from tear PMNs, *p ≤* 0.005), while tear PMNs only showed one peak, at 3 min.

As seen with LPS stimulation, the absolute ROS production (AUC) was larger in tear PMNs compared to that of blood PMNs (Table 2). However, AUC values were similar for both unstimulated and stimulated (as one would expect, the small peak of production at 3 min did not significantly change AUC) tear PMNs, indicating that ROS could be produced, but tear PMNs were unable to mount a full oxidative response following fMLP stimulation.

**Figure 4:**
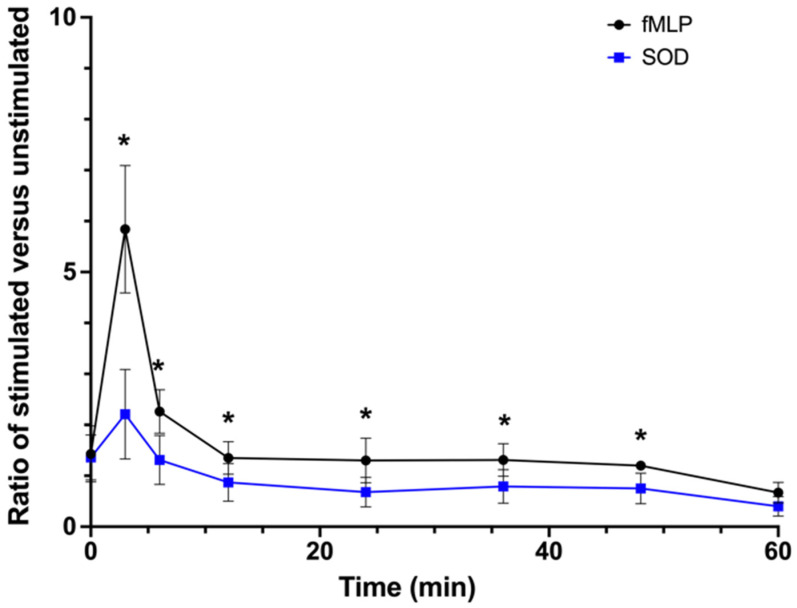
Changes in chemiluminescence of fMLP-stimulated mixed tear PMNs with and without the addition of superoxide dismutase (SOD). Results are reported as ratio of fMLP-stimulated PMNs (with and without SOD) CL emissions over control (unstimulated PMNs) CL emissions. Values are presented as means ± standard deviations; mixed tear PMNs (n = 5). * Significantly different from the ratio of fMLP-stimulated mixed tear PMNs (*p* ≤ 0.029, paired *t* test).

The high and rapid generation of ROS within 3 min after exposure to fMLP has been previously observed in blood PMNs [18,19] and reported to be associated with extracellular production and superoxide anions [15,19]. Superoxide dismutase (SOD) is non-permeable and catalyzes the dismutation of superoxide into either hydrogen peroxide or molecular oxygen. As illustrated in Figure 4, addition of SOD led to a significant decrease in the peak observed at 3 min in fMLP-stimulated mixed tear PMNs (*p* = 0.001). The average reduction in ROS production (AUC) in the presence of SOD was 74 ± 13% (n = 5). These results suggest that, similar to blood PMNs, initial ROS production in fMLP-stimulated tear PMNs is extracellular, and related to NADPH function on the plasma membrane.

### 2.3. ROS Production in PMA-Stimulated PMNs

**Figure 5:**
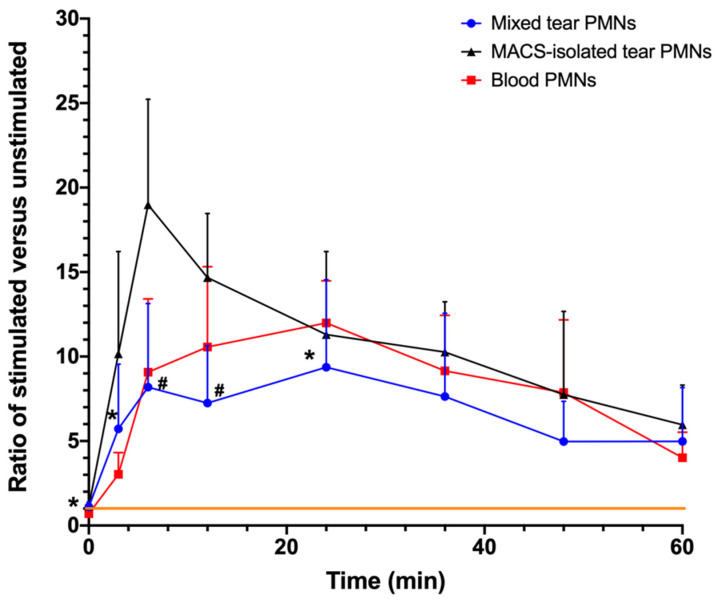
Changes in chemiluminescence (CL) of PMA-stimulated PMNs. Results are reported as ratio of PMA-stimulated PMNs CL emissions over control (unstimulated PMNs) CL emissions. A total of 50,000 cells/well were transferred to wells containing 1 mM luminol and HBSS followed by the addition of 2 μM PMA. Generation of ROS was measured over time using the Cytation 5 at 37 °C. The orange line represents a ratio of 1, meaning no difference between stimulated and unstimulated CL values. Values are presented as means ± standard deviations; mixed tear PMNs (n = 21 from 18 participants), MACS-isolated tear PMNs (n = 10 from 7 participants), and blood PMNs (n = 7 from 4 participants). * Significantly different from the ratio of blood PMNs (*p* ≤ 0.04); # significantly different from the ratio of MACS-isolated tear PMNs (*p* ≤ 0.001).

ROS production in PMNs upon PMA stimulation is shown in Figure 5. Both PMA-stimulated mixed tear PMNs and blood PMNs generated ROS in a continuous manner, with similar trends in kinetics. Mixed tear PMNs had a sharp increase in ROS production around 9 min, and then the amount of ROS produced continuously declined, reaching a plateau around 27 min. There was no significant difference between ROS production of mixed tear PMNs and blood PMNs at all time points (*p* ≥ 0.085) except at 0, 3 and 24 min (*p* ≤ 0.004). Compared to the response to stimulation with fMLP and LPS, mixed tear PMNs showed a higher and prolonged production of ROS upon PMA stimulation.

As seen in Figure 5, large standard deviations in the ROS production of mixed tear PMNs were present due to large differences in individual responses. Two types of responder groups could be identified based on the magnitude of ratio: half of the participants were high responders (HR), with a ratio above 11 (ranging from 11.3 to 48.1) while the other half were low responders with a ratio below 11 (ranging from 4.9 to 9.8).

**Figure 6:**
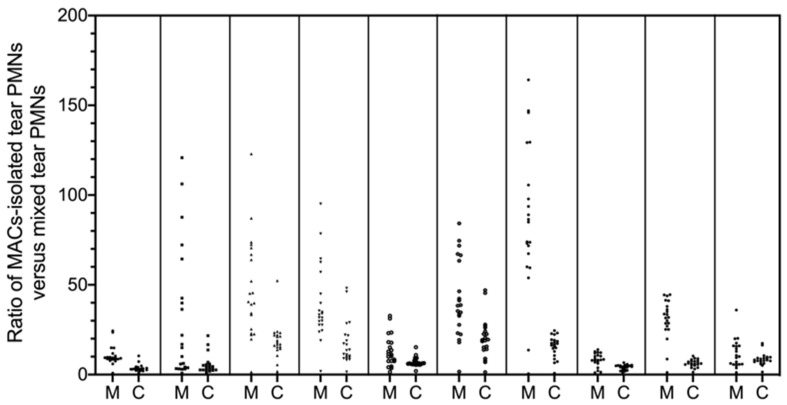
Comparison of ROS production by MACS-isolated tear PMNs and the non-isolated mixed tear PMNs. M: MACS-isolated tear PMNs; C: mixed tear PMNs from eye collection concentrated by centrifugation. Data points represented as the ratios of stimulated versus unstimulated CLU over 60 min at 3 min intervals. n = 10 from 10 different individuals, each presented in individual column.

ROS production induced by PMA stimulation in MACS-isolated tear PMNs led to significantly higher ratios (*p* ≤ 0.011; Figure 5) with broader distribution (Figure 6) when compared to mixed tear PMNs.

As shown in Table 3, as opposed to the results with LPS and fMLP, absolute ROS production (AUC-stimulated) with PMA stimulation significantly increased for both tear and blood PMNs. Despite the large standard deviations observed in the PMA-stimulated PMNs (Figure 5), the relative AUC (AUC for activation ratio curve) of MACS-isolated tear PMNs was significantly higher than that of mixed tear PMNs (*p* = 0.016), which further suggested that MACS-isolated tear PMNs were able to produce more ROS than non-isolated tear PMNs (i.e., mixed tear PMNs). Additionally, the slope between 0 and 15 min was calculated as an indication of the rate of ROS production, and the findings suggested that MACS-isolated tear PMNs could initiate a significantly more rapid ROS response than mixed tear PMNs (*p* = 0.008). The differences observed in ROS production between isolated and non-isolated tear PMNs suggested that tear PMNs may be either primed by the separation process or suppressed by the potential inhibitory/anti-inflammatory interaction with other cells or tear proteins present in the mixed tear PMNs samples.

**Figure 7:**
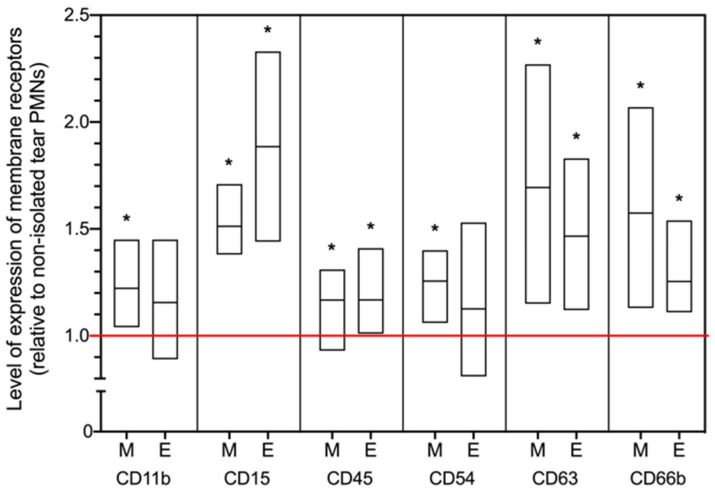
Comparison of the expression of membrane receptors on MACS-isolated or EasySep-isolated tear PMNs with mixed tear PMNs (non-isolated tear PMNs). M: MACS-column cell separation; E: EasySep cell separation. Once cells were collected from the ocular surface, tear PMNs were isolated either by MiniMACS column or the EasySep cell separation system. Samples from the non-isolated cell collection, concentrated by centrifugation, were used as controls. Cells were stained with fluorescently conjugated antibodies and analyzed by flow cytometry. Tear PMNs were identified by side scatter characteristics and CD45. The red line represents a ratio of 1, meaning no difference between the isolated tear PMNs and non-isolated mixed tear PMNs. Values are presented as means ± standard deviations. n = 5 from same 5 participants for each cell separation system. * Significantly different from the non-isolated mixed tear PMNs (*p* ≤ 0.042, paired *t* test).

To determine if the higher ROS generation by MACS-isolated tear PMNs may be due to the potential priming of PMNs during the cell separation process, the phenotype of isolated tear PMNs was compared to non-isolated (i.e., mixed) tear PMNs using flow cytometry. Another cell separation system, EasySep cell separation, was also used to investigate if the difference observed was consistently seen in tear PMNs that were isolated by a microbeads magnetic system. As shown in Figure 7, MACS-isolated tear PMNs showed significantly higher expression of all selected markers of activation compared to non-isolated tear PMNs (*p* ≤ 0.042), while only CD15, CD45, CD63, and CD66b were significantly higher on the EasySep-isolated tear PMNs (*p* ≤ 0.028), suggesting a partial activation of cells by EasySep. The upregulation of the degranulation markers, CD66b and CD63, suggests that activation of tear PMNs may have occurred during the microbead magnetic separation, which would be further supported by the significant upregulation of the adhesion markers CD11b and CD54.

### 2.4. The Phosphorylation State of ERK and p38 MAPK

Since tear PMNs showed differential ROS production when treated with different stimuli, we hypothesized that the signaling molecules within their intracellular signaling pathways or/and the receptors on cell membranes may be impaired. Phosphorylation of p38 MAPK and ERK was assessed by flow cytometry, as these molecules have been reported to play a crucial role in PMA and fMLP-induced cell activation responses [20,21]. Blood PMNs and differentiated HL-60 cells (dHL-60, neutrophil-like cells) were used as positive controls. As shown in Table 4, after stimulation with fMLP, tear PMNs were able to upregulate p38 MAPK and ERK, suggesting the active phosphorylated state of the signaling molecules. Collectively, these results suggest that the intracellular signaling molecules, at least ERK and p38 MAPK, are not depleted and functional in tear PMNs collected upon awakening.

## 3. Discussion

This study provides the kinetic measurements of ROS production during the oxidative (respiratory) burst in tear PMNs in response to LPS, fMLP and PMA using the chemiluminescence assay. The amount of ROS generated is directly correlated to the chemiluminescence (CL) when luminol reacts with ROS and emits light [22]. The CL responses between the tear PMNs (from purified or non-purified cell collection) and the blood PMNs were compared to assess if tear PMNs have the ability to initiate an oxidative burst similar to that of blood PMNs. Each stimulus has different mechanisms of action, which likely explain the distinct CL responses observed in this study and provide further insights on the phenotype of tear PMNs collected after sleep. fMLP and LPS bind to PMNs cell membrane receptors, seven transmembrane spanning GPCRs and TLRs, respectively, to induce various cell functional responses [8,23]. In contrast, PMA permeates into the cell and induce the cell activation by directly binding to PKC [24].

fMLP acts via GPCRs to activate several intracellular signaling pathways involving MAPK (such as p38 MAPK and ERK), phospholipases, and PKC family to trigger various cell responses [8,11,24,25]. While a concentration of less than 10 nM of fMLP can only trigger migration, fMLP concentration larger than 100 nM can induce the oxidative burst [8]. In our study, fMLP at 1.5 μM was able to induce a transient peak of ROS production in tear PMNs within the first three minutes, similarly to what had been observed in blood PMNs. Previous work demonstrated that extracellular ROS is produced by blood PMNs during the first five minutes after fMLP stimulation, while the later ROS/CL response is associated mostly with intracellular production [17,21,26]. Additionally, it has also been reported that the initial ROS released upon activation by fMLP-stimulated blood PMNs is the superoxide anion [18,22]. Our results with and without SOD demonstrated that tear PMNs are able to produce extracellular ROS within the first three minutes of stimulation, indicating that the NADPH oxidase on their cell membranes is fully functional. Furthermore, this also suggest that binding of fMLP to its receptor was able to stimulate phospholipases (PLC and PLD) and produce extracellular ROS through the MAPK pathway and thus there is no potential impairment of fMLP binding to its receptor [11,27,28]. However, tear PMNs appear to be unable to generate the second peak, identified as the intra-cellular ROS response to fMLP [17,26]. The lack of, and therefore the inability of tear PMNs to produce intracellular ROS upon fMLP stimulation may suggest that some intracellular pathways leading to ROS production are dysfunctional or blocked. Under PMA-stimulated conditions, tear PMNs were able to produce ROS, indicating that the downstream PKC signaling pathway was functional, including NADPH oxidase. Thus, the impairment of intracellular ROS production under fMLP-stimulated conditions could be due to (1) alterations in the intracellular signaling pathways or transduction downstream of the fMLP receptors; and/or (2) a reduced number of substrates for phospholipases, which may lead to a shortage for the secondary messengers needed to activate downstream intracellular signaling molecules. The inability to generate ROS due to impaired receptors is unlikely, as discussed above. Based on our results, upon the stimulation of fMLP (and PMA), the expression of ERK and p38 MAPK was upregulated in tear PMNs to a similar extent observed in blood PMNs and dHL-60 cells, meaning that these intracellular signaling molecules could be phosphorylated, and thus that the pathways were functional. Other intracellular signaling molecules, such as phosphoinositide 3-kinase (PI3K) [16], involved in fMLP-induced oxidative bursts, also play key roles, and thus future research may be needed to further investigate the intracellular signaling pathways within tear PMNs in order to gain a better understanding on the regulation of their cellular response to inflammatory stimulus.

In our study, LPS failed to induce ROS production in tear PMNs, with not even an initial 3 min burst of extracellular ROS. TLR-4 is the specific receptor for LPS, which activates the defensive mechanisms of PMNs [29]. TLR-4 defects in PMNs are associated with impaired ability to initiate oxidative bursts in response to LPS [30]. The inability of tear PMNs (clearly observed in both the purified and non-purified PMNs eye wash collections) to produce ROS due to impaired receptor function needs to be further examined.

Under PMA stimulation, PMA diffuses into cells and binds to PKC leading to cell activation. Since PMA triggered an oxidative burst response in tear PMNs, this suggests that the intracellular signaling pathways downstream of PKC are fully functional in tear PMNs. Previous studies using dihydrorhodamine 123 (DHR-123) to measure oxidative burst via flow cytometry showed a limited ability of tear PMNs to generate ROS in response to PMA as compared to blood PMNs [4,7], which appears to contradict our current results. However, our current findings using luminol-enhanced chemiluminescence clearly indicate that ROS production in tear PMNs is time dependent. Flow cytometry can only measure ROS production in a definite point in time, which, depending on how this time point is chosen, may not be appropriate to infer the overall ability of ROS production by tear PMNs. Furthermore, while luminol-enhanced chemiluminescence detects both extra- and intracellular ROS generation [22], DHR-123 can only measure intracellular ROS production in flow cytometry [31]. PMA stimulates both extra-and intracellular ROS generation [10]. Thus, using DHR 123 and flow cytometry might underestimate ROS production unless ROS production is assessed at different time points.

As shown from computing AUC, unstimulated tear PMNs were found to produce more ROS compared to unstimulated blood PMNs. This constitutive or spontaneous ROS level was likely due to their state of activation and their age (having been in the closed eye environment for around 7 h). Unstimulated aging blood PMNs have been reported to release their intracellular pool of ROS that has accumulated from previous activation over an incubation period in vitro [32]. ROS production in the absence of any stimuli has also been reported with the oral neutrophils of healthy individuals, which display signs of activation with high CD11b and CD66b and have higher constitutive ROS levels compared to unstimulated blood PMNs [33]. From receptor upregulation to the presence of NETs, tear PMNs present a phenotype of prior activation in the closed-eye environment [4,6,7]. Higher CL emissions from tear PMNs (29 ± 14, 34 ± 21) compared to blood PMNs (17 ± 4, 13 ± 6), respectively, at 0 and 3 min, in parallel with the higher constitutive ROS production provides further evidence of activation prior to their collection and of their mature stage in the life cycle of PMNs.

The collected eye wash not only contained PMNs, but also low numbers of monocytes and lymphocytes. As monocytes and lymphocytes are able to generate ROS, there was a need to further verify if the ROS measured in this study was produced mainly by tear PMNs. Thus, the MiniMACS-column cell separation system was used to purify tear PMNs. While the purification experiments with isolated tear PMNs confirmed our results with the non-purified cell collection, these experiments also highlighted some interesting facts about the potential of these cells to be primed. Isolated tear PMNs produced higher ROS compared to non-purified/mixed tear PMNs. We hypothesized that this may either be due to the interactions between PMNs and other cells or tear proteins in the eye wash which may suppress their functions, or a priming effect caused by the separation process. By comparing the phenotypes of isolated tear PMNs with mixed tear PMNs (non-purified eye wash cell collection), an enhanced degranulation phenotype (CD66b^+^ and CD63^+^) for both MACS- and EasySep-isolated tear PMNs was observed, a clear indicator of activation that was further confirmed by the upregulation of CD11b and CD54, thus supporting a potential for priming. The activated phenotype on isolated-tear PMNs likely explains their higher ROS production, where elevated levels of CD66b have also been associated with enhanced oxidative responses [34]. Previous results showed that tear PMNs were nonresponsive to in vitro stimulation with fMLP, PMA, CaI, LPS and IL-8 [4,6,35], however, our current results suggest that tear PMNs still have the potential to be activated and/or primed for activation.

Although many questions remain to be answered around ROS production and its mechanisms in tear PMNs, the results of this study have potential translational outcomes around ocular inflammation and clinical research with tear PMNs. The impairment of intracellular ROS production identified with fMLP stimulation and the absence of response to LPS suggest that tear PMNs may be deficient in their ability to generate ROS intracellularly in the phagolysosome after the phagocytosis of bacteria. However, it is also possible that the tear PMNs’ response to stimuli may differ at the end of the night (after prolonged eye closure, when these cells were collected) compared to when they first encounter potential pathogens, and thus further research is required before making a definite conclusion on their phagocytic functionality. While tear PMNs show evidence of activation in the closed-eye environment, stimulation with PMA and the priming/activation induced by the magnetic bead isolation process demonstrated that tear PMNs were not completely exhausted and were able to mount a potent oxidative burst response. This functional potential emphasizes how tear PMNs, the largest population of leukocytes present on the ocular surface under normal/healthy closed-eye conditions, can contribute to ocular defense/homeostasis or ocular inflammation depending on physiological conditions. Understanding how this potential may or may not be triggered during infection may help to develop better approaches to treatment for keratitis and reducing the risks of overnight lens wear. The high level of constitutive ROS production observed over time in vitro, a sign of their more mature stage (after residing in the closed-eye environment for several hours) also suggests that this ROS production took place in the ocular environment. Excessive ROS production or imbalance between oxidants and antioxidants can result in oxidative stress that may damage the host tissues [36]. In healthy eyes, it would appear that this ROS production is part of the homeostatic balance, and the ocular environment is able to protect ocular cells from oxidative stress. Understanding how levels of constitutive ROS production in tear PMNs change in ocular surface diseases (such as dry eye [37]) may contribute a novel direction for therapeutic strategies to reduce ocular inflammation and damage. Given the potential role of tear PMNs in the innate immune response and in ocular inflammation, there is a strong interest and need for more clinical studies to further characterize their phenotypes and perform mechanistic studies that would inform the development of new therapeutic strategies or diagnostic tools [38]. Experimental protocols and the maturity of the collected tear PMNs will need to be carefully considered in result interpretations; our study highlights not only the differential tear PMNs response to stimulation but also how an isolation process (performed in this case using magnetic beads) can affect tear PMNs and their response.

## 4. Materials and Methods

### 4.1. Materials

Endotoxin-free reagents, medium and sterile polypropylene tubes were used in this study to avoid undesired activation of PMNs. LPS (Escherichia coli serotype 0111:B4), PMA, fMLP, Hanks’s balanced salt solution (HBSS), luminol (5-amino-2,3-dihydroxy-1,4-phthalazinedione), and superoxide dismutase (SOD) were ordered from Sigma-Aldrich Co. (Oakville, ON, Canada). Phosphate-buffered saline (PBS) was purchased from Lonza (Allendale, NJ, USA). Dulbecco’s modified eagle media (DMEM) was purchased from Life Technologies (Burlington, ON, Canada). The MiniMACS-column cell separation system was purchased from Miltenyi Biotec Inc. (Auburn, CA, USA). EasySep^TM^ Human CD15 positive selection kit for cell separation was purchased from STEMCELL technologies (Vancouver, BC, Canada). All fluorescently-labelled antibodies were purchased from BD Biosciences (Mississauga, ON, Canada)

### 4.2. Subjects

This study received ethics clearance from the University of Waterloo Human Research Ethics Committee (#30164; Waterloo, ON, Canada) and was conducted in accordance with the tenets of the Declaration of Helsinki. A total of twenty-four healthy non-lens wearer participants, 10 males and 14 females, were recruited in this study, and ages ranged from 20 to 33. All participants provided written informed consent before cell collection.

### 4.3. Tear PMNs Collection and Separation

Participants were trained to collect their cells [4]. Upon awakening, using sterile PBS in a dropper, participants gently washed the ocular surface of each eye, and collected the run-off in a sterile polypropylene tube. Participants were asked to bring their samples to the lab within two hours of collection.

Reconcentrated cell suspension (mixed tear PMNs): the collected eye wash was centrifuged at 280× *g* for 10 min, followed by resuspension in PBS.

MACS-column cell separation system: as per manufacturer’s instructions, the collected eye wash was incubated with CD15 antibody and conjugated with magnetic microbeads in PBS containing 0.5% BSA at 4 °C for 20 min. The MiniMACS-column was placed in the magnetic field of the MACS separator and was rinsed with degassed PBS buffer containing 0.5% BSA three times. Then, the cell suspension was applied to the column. In this isolation procedure, unlabeled cells flowed through the column, while the targeted PMNs (CD15^+^ cells) were retained on the column due to the strong magnetic field. Once the entire cell suspension had been applied to the column, the column was washed three times with degassed PBS buffer. The column was then removed from the separator, and a plunger was used to flush out the targeted cells. The cell suspension was reconcentrated by centrifugation at 280× *g* for 5 min.

EasySep cell separation system: as per manufacturer’s instructions, the collected eye wash was incubated with a CD15 positive selection cocktail for 30 min followed by another 30 min incubation with RapidSpheres^TM^ that contained magnetic particles bound to CD15 antibodies. RoboSep^TM^ buffer containing 2% FBS and 1 mM EDTA was added to dilute the cell suspensions. Then, the tube containing the cell suspension was placed into the magnetic separator for 10 min, followed by removal of the supernatant, which was repeated one more time. The cell suspension was reconcentrated by centrifugation at 280× *g* for 5 min.

After the isolation and reconcentration, cell count and cell viability were assessed using a hemocytometer with Trypan Blue.

### 4.4. Blood PMNs Isolation

Blood was taken from four healthy participants who did not take any anti-inflammatory medication for at least 72 h. Due to the COVID-19 restrictions on research involving human participants, blood samples were not obtained from all participants who provided tear samples. Blood PMNs were isolated using a two-density gradient centrifugation process, using PolymorphPrep (Axis Shield PoC AS, Oslo, Norway) and Histopaque (Oakville, ON, Canada) as described previously [35]. Following isolation, blood PMNs were washed twice in DMEM/10% FBS with 5 mM EDTA and then underwent one last one wash in PBS. A cell count was performed and cells were resuspended in PBS at ~10^5^ cells/mL.

### 4.5. Luminol-Enhanced Chemiluminescence

Chemiluminescence (CL) measurements were assessed on the Cytation 5 Cell Imaging Multi-Mode Reader (BioTek) using 96-well cell culture white microplates (Greiner bio-one, Frickenhausen, Germany). Cells were simulated with PMA (2 μM), fMLP (1.5 μM), and LPS (2 μg/mL) [4], with or without SOD (250 U/mL) which converts the superoxide to either oxygen molecules or hydrogen peroxide. Each well was set to test one stimulus, and each had a total reaction volume of 200 μL containing 50,000 cells, 1 mM luminol, its corresponding stimulus, with or without SOD, and Hanks’ balanced salt solution (HBSS) which was added to a final volume of 200 μL. Controls were included in each experiment and contained the same concentration of reagents but no stimulus (i.e., HBSS was added to complete volume to 200 μL). CL emissions were recorded at 37 °C for 60 min at 3 min intervals.

### 4.6. Antibody Staining and Flow Cytometry

Membrane receptor staining: cell suspensions were stained with fluorescently conjugated antibodies, CD11b, CD15, CD63, CD66b and CD45, for 20 min at room temperature in the dark. Then, cells were diluted with DMEM/10% FBS and were ready for flow cytometry.

Intracellular signaling molecules staining: after cells were stimulated with either 2 μM PMA or 1.5 μM fMLP, they were fixed with pre-warmed 4% formaldehyde at 37 °C for 10 min. Then, cells were pelleted by centrifugation, followed by two washes with perm/wash buffer (BD Bioscience) at 1200 rpm for 5 min. Cells were stained with anti-ERK, anti-p38 MAPK antibodies, and CD45 for 30 min in the dark at room temperature. After another wash with perm/wash buffer, cells were resuspended in DMEM/10% FBS and were ready for flow cytometry.

The PMN population was identified based on double gating the cells that were high in side-scattered light (granularity), low in forward-scatter light (size), and CD45^+^. All samples were acquired on a Becton Dickinson FACS Calibur flow cytometer (Mountain View, CA, USA) using CELLQuest software (Becton Dickinson, Mountain View, CA, USA). At least 2000 PMN events were acquired, and fluorescent values for each antibody (also known as Mean Fluorescent Intensities, MFI) were recorded for all samples.

### 4.7. Statistics

After analysis of the CL kinetic graphs and based on peak responses, statistical analysis was performed on the CL response at 0, 3, 6, 12, 24, 36, 48, 60 min. The ratio of stimulated over control CL emissions (CL_stimulated_/CL_control_) were calculated, and are reported as means ± standard deviations in function of time. GraphPad Prism software (GraphPad Software, San Diego, CA, USA) was used to compute rate and area under the curve (AUC). A Welch’s *t*-test and paired *t* test were performed using IBM SPSS software (IBM Canada Ltd., Markham, ON, Canada), and a *p*-value of less than 0.05 was required for statistical significance.

## Figures and Tables

**Table 1:** Absolute and relative ROS production in unstimulated and LPS-stimulated PMNs, as measured by AUC.

	Mixed Tear PMNs	MACS-Isolated Tear PMNs	Blood PMNs
AUC Unstimulated	10,803 ± 3810	6171 ± 4475	4384 ± 2275
AUC LPS-stimulated	11,048 ± 5075	5366 ± 4412	6659 ± 3262
AUC Activation ratio	18 ± 11	17 ± 12	39 ± 27

AUC (area under the curve) was computed using GraphPad Prism software. n = 12 from 10 participants for mixed tear PMNs, n = 6 from 6 participants for MACS-isolated tear PMNs, and n = 5 from 4 participants for blood PMNs; values are represented as means ± standard deviations.

**Table 2:** Absolute and relative ROS production in unstimulated and fMLP-stimulated PMNs, as measured by AUC.

	Mixed Tear PMNs	MACS-Isolated Tear PMNs	Blood PMNs
AUC Unstimulated	8943 ± 4389	5210 ± 4375	4384 ± 2276
AUC fMLP-stimulated	10,489 ± 4475	7264 ± 5444	8529 ± 3296 ^#^
AUC Activation ratio	35 ± 14 *	50 ± 26 *	70 ± 15

AUC (area under the curve) were computed using GraphPad Prism Software. n = 16 from 15 participants for mixed tear PMNs, and n = 11 from 9 participants for MACS-isolated tear PMNs, and n = 5 from 4 participants for blood PMNs, values are represented as means ± standard deviations. * Significantly different from blood PMNs (*p* ≤ 0.01). # Significantly different from unstimulated PMNs (*p* ≤ 0.005, paired *t* test).

**Table 3:** Absolute and relative ROS production in unstimulated and PMA-stimulated PMNs, as measured by AUC.

	Mixed Tear PMNs	MACS-Isolated Tear PMNs	Blood PMNs
AUC Unstimulated	8204 ± 4145	6384 ± 2256	4384 ± 2276
AUC PMA-stimulated	40,640 ± 13,529 ^#^	50,819 ± 26,052 ^#^	35758 ± 15365 ^#^
AUC Activation ratio	552 ± 454 *	1910 ± 1604	473 ± 308 *
Slope	0.62 ± 0.71 *	3.49 ± 2.96	0.67 ± 0.35 *

AUC and slope of mixed tear PMNs and MACS-isolated PMNs were computed using GraphPad Prism software. n = 21 from 18 participants for mixed tear PMNs, n = 10 from 7 participants for MACS-isolated tear PMNs, and n = 7 from 4 participants for blood PMNs; values are represented as means ± standard deviations. * Significantly different from MACS-isolated tear PMNs, *p* ≤ 0.015. ^#^ Significantly different from unstimulated PMNs (*p* ≤ 0.001, paired *t* test).

**Table 4:** Changes in the expression of ERK and p38 MAPK on tear PMNs, blood PMNs and dHL-60 cells in response to fMLP and PMA.

	Mixed Tear PMNs		Blood PMNs		dHL-60 Cells	
	fMLP	PMA	fMLP	PMA	fMLP	PMA
ERK	1.59 ± 0.08	1.26 ± 0.08	1.30 ± 0.09	1.16 ± 0.04	1.28 ± 0.03	1.39 ± 0.20
p38 MAPK	1.53 ± 0.24	1.18 ± 0.05	1.41 ± 0.16	1.19 ± 0.16	1.33 ± 0.04	1.42 ± 0.08

Results are shown as ratio of stimulated versus unstimulated cells; values are represented as means ± standard deviations. n = 3 for each blood PMNs, mixed tear PMNs, and dHL-60 cells.

## Data Availability

The datasets analyzed during the current study are available from the corresponding author upon reasonable request.
